# Dynamic Expression of Serotonin Receptor 5-HT3A in Developing Sensory Innervation of the Lower Urinary Tract

**DOI:** 10.3389/fnins.2016.00592

**Published:** 2017-01-06

**Authors:** K. Elaine Ritter, E. Michelle Southard-Smith

**Affiliations:** Division of Genetic Medicine, Department of Medicine, Vanderbilt University School of MedicineNashville, TN, USA

**Keywords:** serotonin, neural crest, autonomic nervous system, lower urinary tract, dorsal root ganglia

## Abstract

Sensory afferent signaling is required for normal function of the lower urinary tract (LUT). Despite the wide prevalence of bladder dysfunction and pelvic pain syndromes, few effective treatment options are available. Serotonin receptor 5-HT3A is a known mediator of visceral afferent signaling and has been implicated in bladder function. However, basic expression patterns for this gene and others among developing bladder sensory afferents that could be used to inform regenerative efforts aimed at treating deficiencies in pelvic innervation are lacking. To gain greater insight into the molecular characteristics of bladder sensory innervation, we conducted a thorough characterization of *Htr3a* expression in developing and adult bladder-projecting lumbosacral dorsal root ganglia (DRG) neurons. Using a transgenic *Htr3a*-EGFP reporter mouse line, we identified 5-HT3A expression at 10 days post coitus (dpc) in neural crest derivatives and in 12 dpc lumbosacral DRG. Using immunohistochemical co-localization we observed *Htr3a*-EGFP expression in developing lumbosacral DRG that partially coincides with neuropeptides CGRP and Substance P and capsaicin receptor TRPV1. A majority of *Htr3a-EGFP*+ DRG neurons also express a marker of myelinated Aδ neurons, NF200. There was no co-localization of 5-HT3A with the TRPV4 receptor. We employed retrograde tracing in adult *Htr3a*-EGFP mice to quantify the contribution of 5-HT3A+ DRG neurons to bladder afferent innervation. We found that 5-HT3A is expressed in a substantial proportion of retrograde traced DRG neurons in both rostral (L1, L2) and caudal (L6, S1) axial levels that supply bladder innervation. Most bladder-projecting *Htr3a-EGFP*+ neurons that co-express CGRP, Substance P, or TRPV1 are found in L1, L2 DRG, whereas *Htr3a*-EGFP+, NF200+ bladder-projecting neurons are from the L6, S1 axial levels. Our findings contribute much needed information regarding the development of LUT innervation and highlight the 5-HT3A serotonin receptor as a candidate for future studies of neurally mediated bladder control.

## Introduction

The lower urinary tract (LUT), comprised of the bladder and urethra, relies on autonomic and sensory neural input for storage and appropriate expulsion of urine from the body. Damage to or degeneration of any neural component of this system can lead to chronic pelvic pain or bladder dysfunction, which can severely diminish patient quality of life. Additionally, sensitization of sensory components that originate within the dorsal root ganglia can occur in response to bladder inflammation or chronic interstitial cystitis. Current treatment options for these conditions tend to affect many diverse neuronal populations and thus have numerous adverse side effects. Better understanding of the molecular features of bladder-innervating sensory afferents, and how they develop, has the potential to inform more pharmacologically specific treatment options for these conditions.

Afferent fibers supplying the LUT express a wide variety of ion channels and neuropeptides with known roles in mediating mechanosensation of bladder fullness and nociceptive processing of painful stimuli (de Groat and Yoshimura, 2009). Many of these markers, such as TRPV1 and TRPV4, have been well-studied. Surprisingly, serotonin signaling has not been as thoroughly investigated in the LUT, despite its known importance in sensory processing in other systems. Serotonin receptors are expressed in both the central and peripheral nervous systems and are involved in every level of nociception, from the peripheral site of injury or inflammation to cognitive perception of pain (Gold and Gebhart, 2010). Serotonin can exert both pronociceptive and antinociceptive effects, depending on the receptors activated in peripheral tissues and in the spinal cord. Of the 14 different serotonin receptor subtypes, 5-HT3A (encoded by the *Htr3a* gene) is known to be an especially important mediator of nociception (Zeitz et al., 2002; Kayser et al., 2007), including visceral pain. Specifically, antagonizing 5-HT3A alleviates pain associated with intestinal inflammation and irritable bowel syndrome (Chen et al., 2009; Walstab et al., 2010; Machu, 2011). Additionally, normal 5-HT3A receptor activity is required for adult LUT innervation and bladder function (Bhattacharya et al., 2004). However, it remains unclear what sensory subtypes normally express the 5-HT3A receptor and the extent to which 5-HT3A+ neurons contribute to normal bladder innervation.

Specification of the sensory neuronal lineage from neural crest progenitors occurs between 9.5 and 11 days post coitus (dpc) as neurogenesis progresses. Differentiation of DRG neurons and acquisition of sensory subtype-specific markers has been reported around 14.5 dpc (Marmigerè and Ernfors, 2007; Bachy et al., 2011; Zou et al., 2012). Despite evidence that points to an effect of 5-HT3A on bladder function (Espey et al., 1998; Bhattacharya et al., 2004; Hall et al., 2015), no prior studies have assessed the expression of this receptor in DRG during the developmental stages when the LUT is being innervated. To date, *Htr3a* gene expression in DRG has only been characterized via *in situ* hybridization at a single developmental time point (Tecott et al., 1993; Diez-Roux et al., 2011). Using a transgenic *Htr3a*-EGFP mouse line in conjunction with immunohistochemistry and retrograde tracing, we characterized the developmental expression patterns of 5-HT3A in DRG and the contribution of neurons expressing this receptor to adult urinary bladder sensory innervation. We find that this serotonin receptor exhibits dynamic expression patterns over the course of sensory neuron development and contributes to the majority of bladder innervation.

## Materials and methods

### Animals

All experimental protocols were approved by the Vanderbilt University Institutional Animal Care and Use Committee (IACUC). Tg(*Htr3a*-EGFP)DH30Gsat/Mmnc (Stock Number 000273-UNC) transgenic mice were obtained from the Mutant Mouse Resource & Research Center at the University of North Carolina. *Htr3a*-EGFP mice were maintained as heterozygotes on an outbred Swiss Webster background (Taconic). All animals were provided food and water *ad libitum* and kept on a 14-h on, 10-h off light cycle. To obtain tissues at specific fetal stages, males and female mice were paired for overnight matings and the morning of observing a seminal plug was designated as 0.5 days post coitus (dpc).

### Tissue processing

#### Dissection

Harvested mouse fetuses were collected into ice-cold 1X Phosphate Buffered Saline (PBS). All fetuses and micro dissected tissues were fixed in 10% Neutral Buffered Formalin (NBF, Sigma Aldrich HT501128). Younger fetuses (10, 11, and 12 dpc) were fixed intact for 6 h at 4°C. Older fetuses, 14 and 18 dpc, were further sub-dissected to allow permeation of fixative to visceral tissues, then fixed overnight at 4°C. Because the DRG in P2 pups are small and fragile, “backbone blocks” were dissected from axial levels T13 to S4 and fixed intact overnight at 4°C. P14, P28, and P90 DRG were sub-dissected and fixed for 3 h at 4°C. Following fixation, all tissues were washed in cold 1XPBS 3 times for 15 min with a final 1 h wash. Tissues for cryo-sectioning were infiltrated with 30% sucrose in 1XPBS and stored in the same solution at 4°C until the day of embedding and sectioning.

#### Immunohistochemistry staining

Tissues were embedded in Tissue Freezing Medium (TFM, General Data, #TFM) and immediately sectioned in a Leica Cryostat (CM1900-UV). Sagittal sections 20 μm thick were mounted onto slides treated with 3-APES (Sigma-Aldrich, A3648). For the purpose of cell counting in adult DRGs, every fifth section was mounted to ensure a minimum gap of 100 μm between sections to avoid double-counting cells. Sections on slides were dried on a 37°C slide warmer for 30 min and protected from light. Slides were then immersed in 1XPBS-0.3% Triton-X100 for 5 min at room temperature to remove TFM and permeabilize the tissue for improved antibody penetration. Blocking solution comprised of 1XPBS-0.3%TritonX-100, 10% Bovine Serum Albumin (Sigma-Aldrich, A2153), and 5% Normal Donkey Serum (Jackson ImmunoResearch, 017-000-121, RRID AB_2337258) was applied to sections for a minimum of 30 min at room temperature. The same solution was used to dilute the primary and secondary antibodies. All antibodies used in this study have been thoroughly characterized and validated in knockout mouse lines, as summarized in Tables 1, 2 (Cao et al., 1998; Baiou et al., 2007; Gevaert et al., 2007; Glaser et al., 2007; Cassereau et al., 2013). Blocking solution was tipped off the slides, and diluted primary antibody incubated on sections overnight at 4°C. On the following day, sections were rinsed with sterile 1XPBS and incubated in secondary antibody for 1 h at room temperature. After rinsing, 0.5 mM cupric sulfate in 50 mM ammonium acetate buffer (pH 5.0) was applied to tissue sections for 10 min to quench autofluorescence (Potter et al., 2012). Finally, a gentle rinse with sterile water was used to stop the CuSO_4_ quenching reaction. The slides were mounted and coverslipped with AquaPolyMount (PolySciences, Inc., 18606), and imaged using a Zeiss LSM 510Meta confocal microscope.

**Table 1:** **Primary Antibodies Used in Immunohistochemistry Experiments**.

**Antibody**	**Host**	**Vendor**	**RRID**	**Dilution**	**Validation in Knockout Mouse Line**
CGRP	Rabbit	Sigma Aldrich, #C8198	AB_259091	1:1000	Glaser et al., 2007
Substance P	Rabbit	ImmunoStar, #20064	AB_572266	1:500	Cao et al., 1998
TRPV1	Rabbit	Neuromics, #RA14113	AB_2194034	1:500	Baiou et al., 2007
TRPV4	Rabbit	Alomone Labs, #ACC-034	AB_2040264	1:1000	Gevaert et al., 2007
NF200	Rabbit	Sigma Aldrich, #N4142	AB_477272	1:500	Cassereau et al., 2013
Hu	Human	Vanda Lennon, Mayo Clinic	N/A	1:10,000	N/A

*All primary antibodies used, except for Hu, have been validated in knockout mouse lines*.

**Table 2:** **Secondary antibodies used in immunohistochemistry experiments**.

**Antibody**	**Vendor**	**RRID**	**Dilution**
Donkey anti-Rabbit Cy3	Jackson ImmunoResearch, #711-165-152	AB_2307443	1:1000
Donkey anti-Human Cy5	Jackson ImmunoResearch, #709-605-149	AB_2340578	1:200

### Retrograde tracing of bladder-innervating neurons

#### Retrograde tracing surgery

Adult *Htr3a*-EGFP mice at 12 weeks of age were used for retrograde tracing experiments. Male mice were used exclusively to avoid confounding effects of estrous cycle on neuronal gene expression patterns (Mónica Brauer and Smith, 2015). Injection of retrograde tracing dye, Fast Blue, into the bladder dome was carried out under general anesthesia as previously described (Payne et al., 2015). Briefly, mice were fully anesthetized via 4% isoflurane inhalation and kept on a heating pad for the duration of the surgery. An abdominal incision through the skin and muscle was made to reveal the bladder, which was then gently exteriorized and moistened with sterile saline solution. Fast Blue retrograde tracing dye (PolySciences, Inc., 17740-1) was injected in 9 different locations in 0.5 μL volumes: three sites near the bladder neck, three sites around the middle circumference of the bladder, and three sites near the apex of the bladder dome. A Hamilton syringe equipped with a 33G needle was used for dye injections to avoid bleeding and bladder tissue damage (Hamilton Company #7803-05). Sterile cotton swabs and surgical grade sterile saline were used to carefully remove any excess dye leaking from each injection site. To avoid any dye leakage from the injection sites, sterile cotton swabs and surgical grade sterile saline were used to carefully blot and wash away any excess dye. The bladder was then returned to the abdominal cavity, and the muscle and skin were subsequently sutured. Mice were treated with pre-operative and post-operative analgesic for pain management (buprenex, 0.1 mg/kg, Patterson Veterinary Supply 12496075705). To permit transport of dye back to the neuron somata in the DRG, mice were euthanized on the 7th day following dye tracer injection. Dorsal root ganglia were sub-dissected and processed as described above.

#### Cell counting

Images were captured via confocal microscopy using an Olympus FV-1000 inverted confocal microscope. Images were then exported from the FluoView viewing software as.tiff files and assembled in Adobe Photoshop (2014 2.2 release, Adobe Systems Inc.). Due to heterogeneity in expression intensity of the *Htr3a*-EGFP transgene, images were minimally adjusted for optimal brightness and contrast. Numbers of neurons (Hu+ cells), *Htr3a*-EGFP+ cells, Fast Blue+ cells, and cells labeled with markers of sensory neurons were manually quantified by visual inspection of assembled confocal stacks. Proportions of neuronal subtypes were calculated as the number of immuno-positive cells for each marker over the total number of Hu+ neurons for each section. Cells were counted from three to six sections of three DRG from each axial level group from four *Htr3a*-EGFP animals.

#### Data analysis

One-way ANOVA was conducted to examine differences in average *Htr3a*-EGFP and Fast Blue proportions between all axial levels examined (L1,L2; L3-L5; L6,S1). Differences in *Htr3a*-EGFP and Fast Blue proportions between specific axial level groups were analyzed by Welch's *t*-test. *P*-values were corrected for multiple comparisons; *p*-values less than 0.05 were considered to be statistically significant. Proportions of neuronal subtype markers between lumbar and sacral axial levels were analyzed by Welch's *t*-test; *p*-values less than 0.05 were considered to be statistically significant.

## Results

### *Htr3a*-EGFP is expressed early in sensory nervous system development

**Figure 1:**
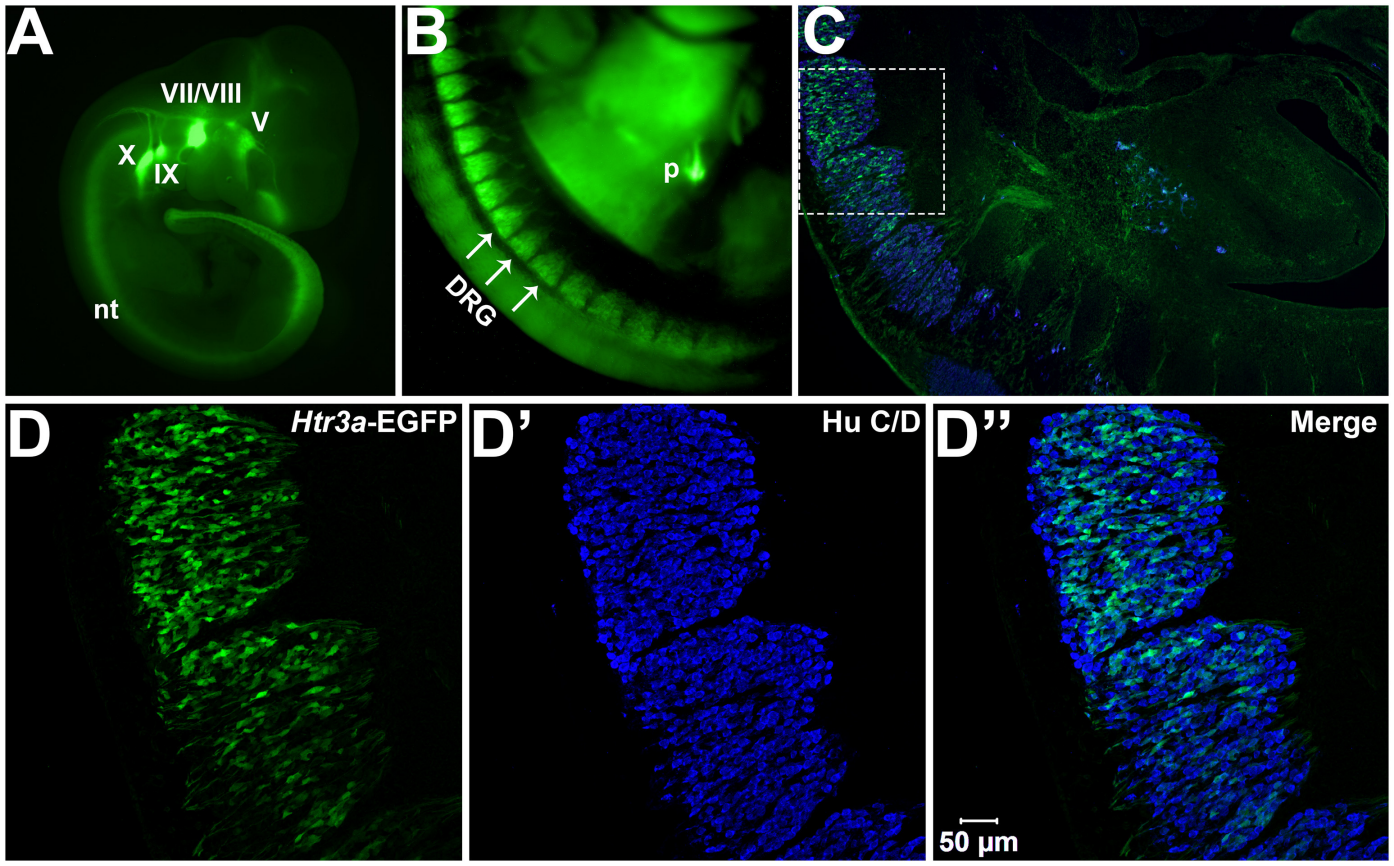
***Htr3a* is expressed early in fetal development of sensory neurons. (A)** Lateral whole-mount image of an *Htr3a*-EGFP transgenic fetus age 10 days post coitus (dpc). EGFP fluorescence is visible in the cranial ganglia (V, VII/VIII, IX, X) and neural tube (nt). **(B)** Lateral Whole-mount view of an *Htr3a*-EGFP 12 dpc mouse fetus at the trunk axial level between the forelimbs and hindlimbs. *Htr3a*-EGFP transgene expression is evident in the dorsal root ganglia (DRG) and pancreas (p). **(C)** Sagittal cryo-section of *Htr3a*-EGFP 12 dpc fetus, stained with Hu to label all neurons (blue). EGFP expression is observed in the lumbosacral DRG. **(D–D”)** 200x magnification of two sacral DRG seen in **(C)**. Counter-staining with Hu C/D allows comparison of EGFP fluorescence to the total neuronal population.

We first sought to define the time point at which *Htr3a* expression initiates during early phases of DRG neurogenesis. To visualize *Htr3a* gene expression, we employed the Tg (*Htr3a*-EGFP) DH30Gsat BAC transgenic reporter mouse line, hereafter referred to as *Htr3a*-EGFP. Validation of this reporter line as an accurate readout of endogenous gene expression has been performed by other groups (Gong et al., 2003; Vucurovic et al., 2010). Using *Htr3a*-EGFP transgenic tissues, the earliest stage at which we observed transgene expression by whole mount imaging was 10 dpc, where strong EGFP fluorescence was evident in the neural crest derived cranial ganglia and within the neural tube (Figure 1A). Hu C/D, a pan-neuronal marker, was strongly expressed in DRG of 11 dpc fetuses along their entire length; however, *Htr3a*-EGFP expression in the DRG was restricted to cervical and upper thoracic axial levels at this stage (data not shown). We first observed *Htr3a*-EGFP transgene expression in lumbosacral DRG that are known to provide bladder innervation at 12 pdc (Figures 1B,C). At this stage *Htr3a*-EGFP expression was observed in a rostral to caudal gradient among maturing DRG (Figures 1B,C) within a subset of Hu+ differentiated neurons (Figures 1D–D”). The rostral vs. caudal difference in *Htr3a*-EGFP expression intensity was prominent, with many neurons exhibiting *Htr3a*-EGFP in one lumbosacral DRG while few cells express the transgene in an immediately adjacent lumbosacral DRG. Additionally, at 12 dpc neuronal processes extending from lumbosacral DRG toward the urogenital sinus and pelvic region are visibly labeled by EGFP (Figure 1C). These experiments show that the 5-HT3A receptor is expressed early in neural crest-derived (Hu+) neurons and is first expressed in lumbosacral DRG by 12 dpc.

### *Htr3a*-EGFP co-localizes with neuropeptides CGRP and substance P in a subset of DRG neurons

**Figure 2:**
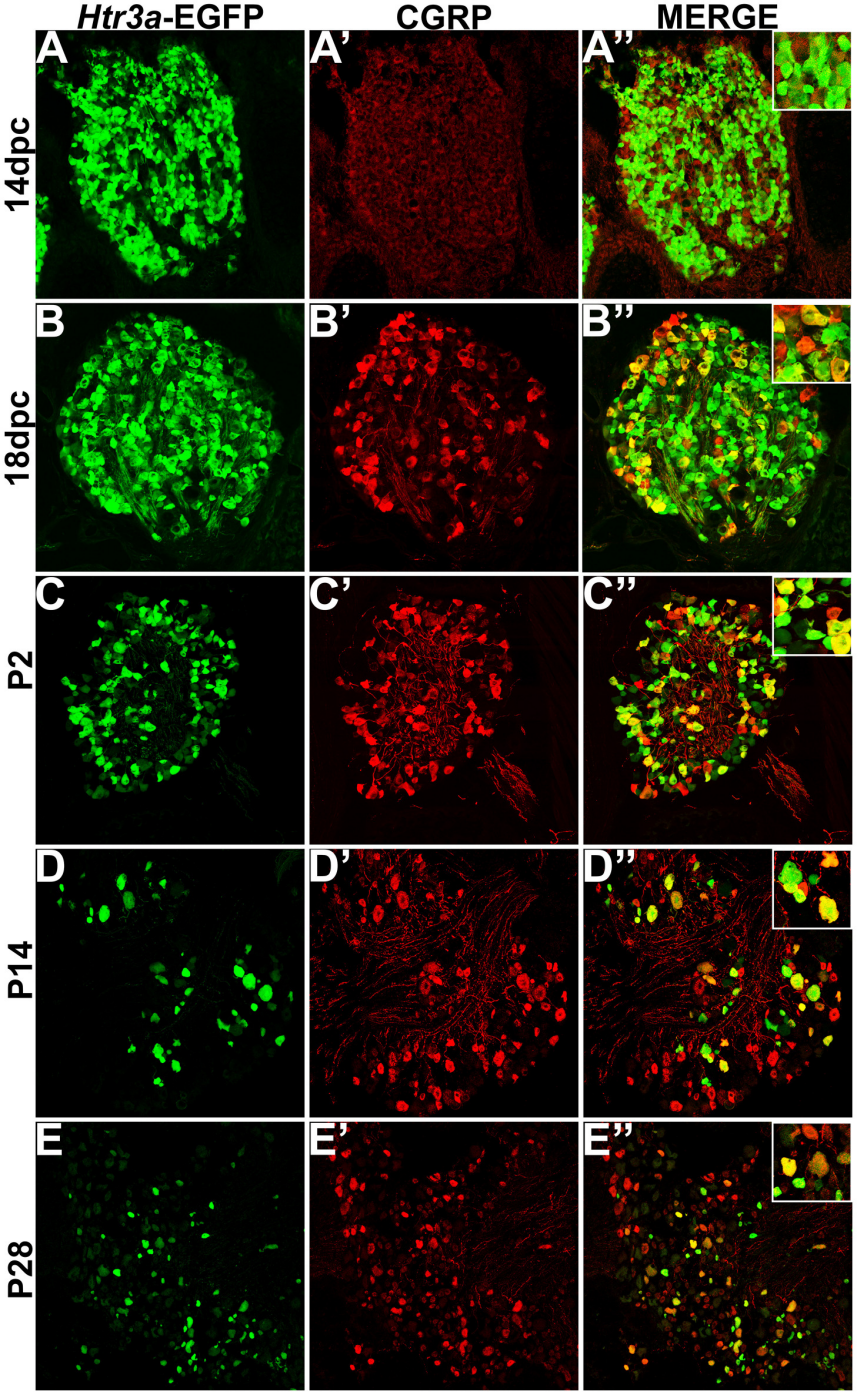
***Htr3a*-EGFP and Calcitonin Gene Related Peptide (CGRP) are co-expressed in a subset of neurons through development and adulthood**. Confocal images of cryosections from *Htr3a*-EGFP transgenic animals stained with anti-CGRP are shown. All DRG presented are from lumbosacral axial levels. **(A–A”)** Sagittal section of 14 dpc fetal DRG. **(B–B”)** Sagittal section of 18 dpc fetal DRG. **(C–C”)** Coronal section of P2 DRG. **(D–D”)** Cryosection of P14 male DRG. **(E–E”)** Cryosection of P28 male DRG. All zoom insets are 400X.

Calcitonin Gene Related Peptide (CGRP) and Substance P (SP) have well-established roles in modulating adult LUT function and pain processing (Laird et al., 2000; Saban et al., 2000; Kiss et al., 2001; Lagerström et al., 2011; Russell et al., 2014) and are normally produced by unmyelinated Aδ nociceptive neurons (Arms and Vizzard, 2011). However, the expression patterns of these neuropeptides in developing mouse lumbosacral DRG projecting to the bladder are unknown. Using immunohistochemistry on *Htr3a*-EGFP+ transgenic tissues, we sought to define co-expression patterns of nociceptive neuropeptides and *Htr3a* in developing lumbosacral DRG. At stage 14 dpc, *Htr3a*-EGFP was strongly expressed in the majority of lumbosacral DRG neurons; however, CGRP expression was negligible at this stage (Figures 2A–A”). By 18 dpc, CGRP was clearly present with a continuous gradient of expression intensity that ranged from moderate to strong between individual cells. Immunostaining for CGRP was observed to co-localize with *Htr3a*-EGFP fluorescence in a subset of large-diameter neurons (Figures 2B–B”). We observed that *Htr3a*-EGFP transgene expression gradually becomes restricted to a subset of cells during the course of postnatal development. This restriction became notable as early as postnatal day (P)2 and was also prevalent at P14 and P28. Specifically, as DRG development progressed, the number of neurons expressing *Htr3a*-EGFP diminished. Among the neurons that remained *Htr3a*-EGFP+, there was considerable heterogeneity in expression intensity, with some cells exhibiting bright EGFP fluorescence while others are substantially dimmer. Despite the gradual restriction of *Htr3a*-EGFP expression and the heterogeneity in expression intensity between individual neurons, partial co-localization with CGRP was maintained through postnatal development and into adulthood (Figures 2C–E”).

**Figure 3:**
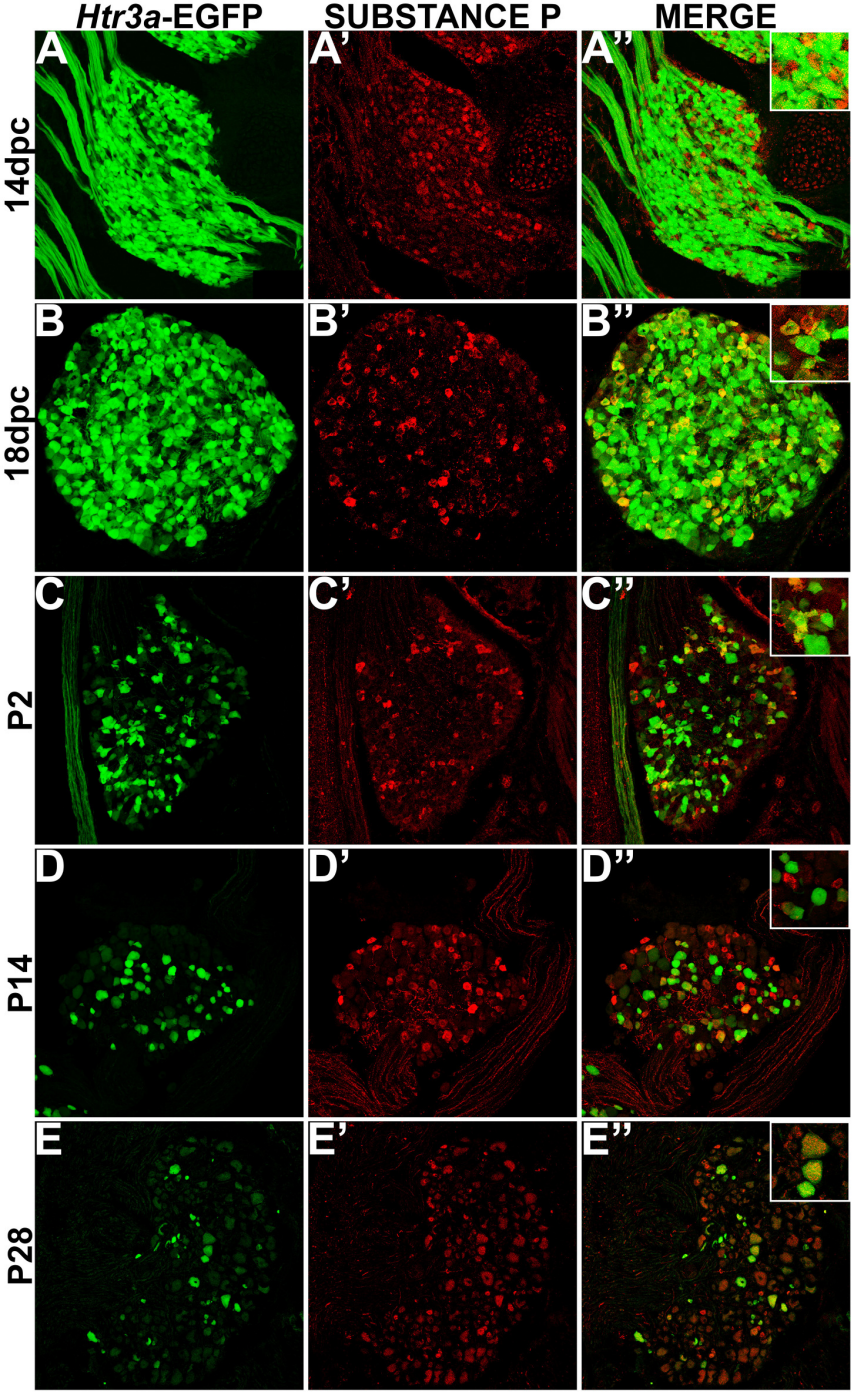
**Some, but not all, *Htr3a*-EGFP neurons express Substance P neuropeptide**. Confocal images of cryosections from *Htr3a*-EGFP transgenic animals stained with anti-Substance P are shown. All DRG presented are from lumbosacral axial levels. **(A–A”)** Sagittal section of 14 dpc fetal DRG. **(B–B”)** Sagittal section of 18 dpc fetal DRG. **(C–C”)** Coronal section of P2 DRG. **(D–D”)** Cryosection of P14 male DRG. **(E–E”)** Cryosection of P28 male DRG. All zoom insets are 400X.

While CGRP and SP are often co-expressed in afferent neurons, other evidence suggests that these neuropeptides do not always overlap and have functionally distinct roles in nociception (Su et al., 1986; Kestell et al., 2015). Given this information we examined co-expression patterns of SP and *Htr3a*-EGFP. Upon staining for SP, we noted faint and diffuse expression throughout the ganglion and partial overlap with *Htr3a*-EGFP transgene fluorescence at 14 dpc (Figures 3A–A”). Co-localization by immunohistochemical staining of 18 dpc DRG for SP revealed that the majority of SP+ neurons also expressed *Htr3a*-EGFP (Figures 3B–B”). However, shortly after birth at P2, co-localization of *Htr3a*-EGFP and SP lessened as *Htr3a*-EGFP developed a heterogeneous pattern of expression intensity among individual neurons within the ganglion (Figures 3C–C”). Substance P staining in postnatal DRG was more uniform; all SP+ neurons exhibited a similar level of expression intensity. At 2 and 4 weeks after birth, only a subset of DRG neurons co-expressed *Htr3a*-EGFP and SP (Figures 3D–E”). From these experiments we conclude that fetal lumbosacral DRG neurons widely co-express 5-HT3A and the neuropeptides CGRP and Substance P, and as postnatal maturation of the DRG occurs, expression of these markers becomes refined to a subpopulation of neurons.

### The majority of TRPV1+ neurons co-express *Htr3a*-EGFP

**Figure 4:**
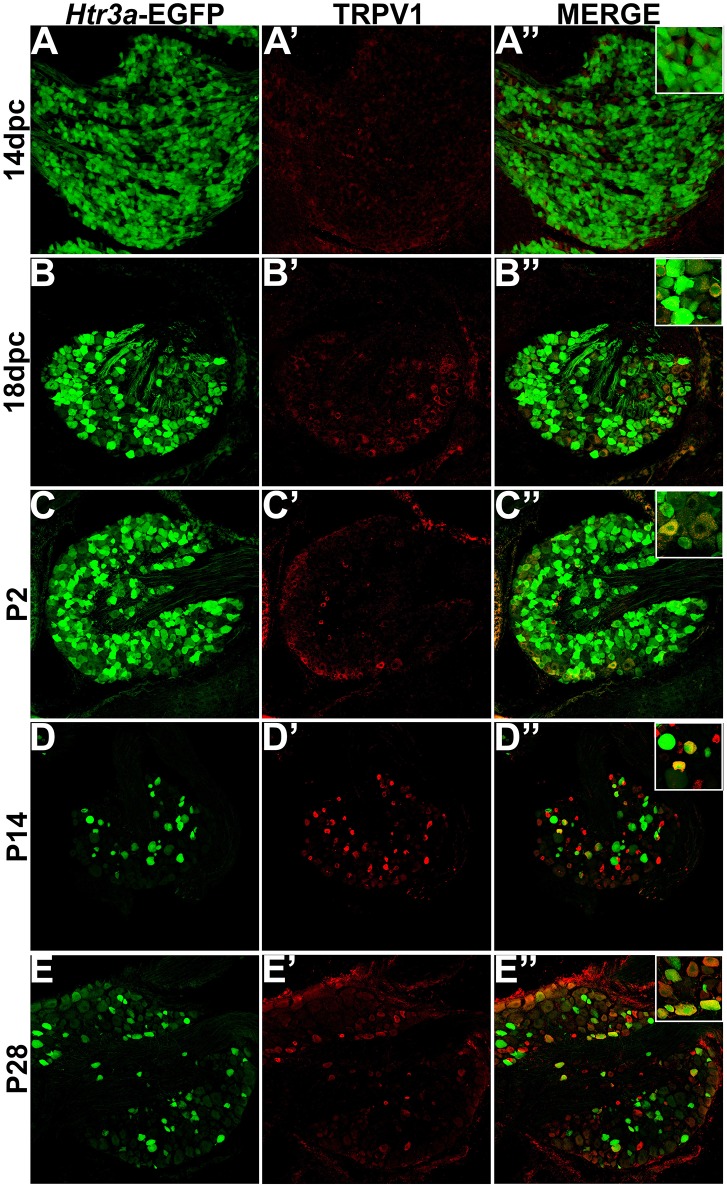
**The majority of adult TRPV1+ neurons also express *Htr3a*-EGFP**. Confocal images of cryosections from *Htr3a*-EGFP transgenic animals stained with anti-TRPV1 are shown. All DRG presented are from lumbosacral axial levels. **(A–A”)** Sagittal section of 14 dpc fetal DRG. **(B–B”)** Sagittal section of 18 dpc fetal DRG. **(C–C”)** Coronal section of P2 DRG. **(D–D”)** Cryosection of P14 male DRG. **(E–E”)** Cryosection of P28 male DRG. All zoom insets are 400X.

Transient Receptor Potential (TRP) channels are necessary for normal adult bladder function (Birder et al., 2002; Arms and Vizzard, 2011; Yoshiyama et al., 2015). Despite the known physiological interaction of 5-HT3A and TRPV1 receptors in other sensory ganglia (Loyd et al., 2011; Kim et al., 2014), the co-localization of TRPV1 with *Htr3a* has not previously been examined during development. To address this gap in knowledge we conducted immunostaining on *Htr3a*-EGFP lumbosacral DRG for TRPV1. At 14 dpc we found diffuse, very low expression of the TRPV1 receptor (Figures 4A–A”). By 18 dpc, TRPV1 expression was weak and diffuse throughout the ganglion, with only a few cells exhibiting strong expression. Of the TRPV1+ cells at 18 dpc, nearly all of them co-express *Htr3a*-EGFP (Figures 4B–B”). TRPV1 expression increased in a subset of neurons by P2 at the same time that *Htr3a*-EGFP became less widespread and began to show heterogeneous levels of EGFP fluorescence among individual neurons (Figures 4C–C”). Heterogeneous expression of the *Htr3a*-EGFP transgene and partial co-localization with TRPV1 was consistently observed at P14 and P28 (Figures 4D–E”). From these experiments we conclude that as DRG neuronal populations mature, there is substantial overlap of *Htr3a* and TRPV1 expression, but lumbosacral DRG also exhibit many *Htr3a*+;TRPV1- neurons.

### *Htr3a*-EGFP does not co-localize with TRPV4

**Figure 5:**
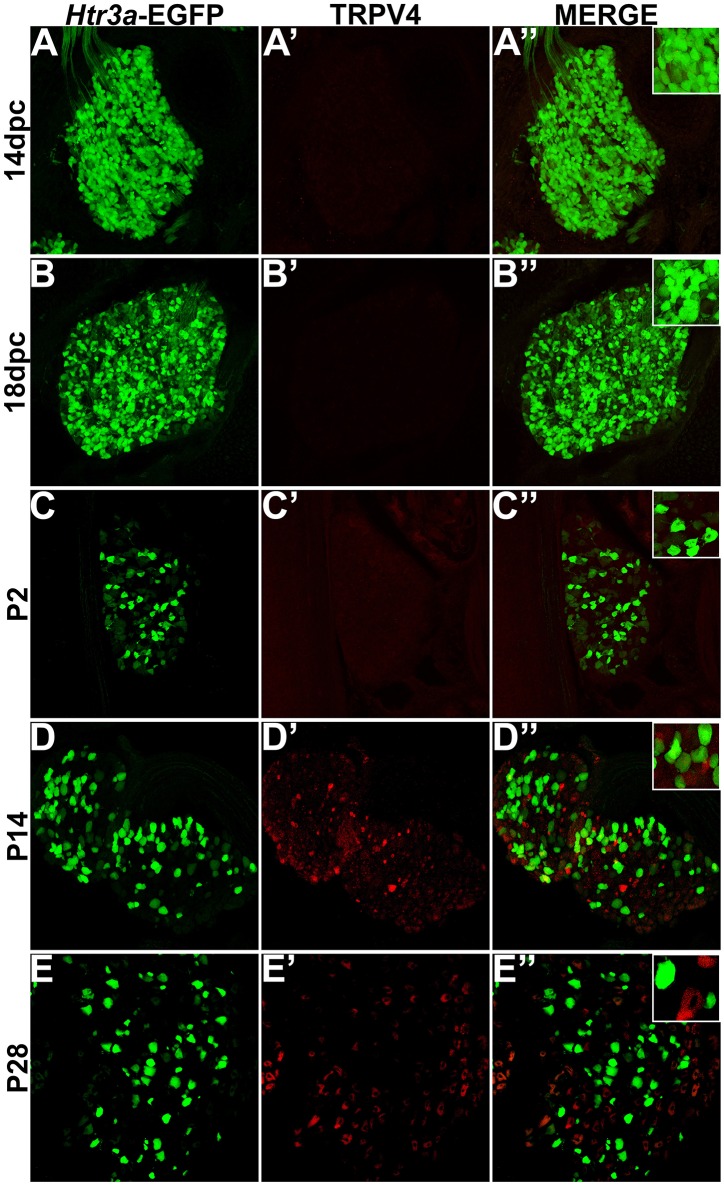
***Htr3a*-EGFP does not co-localize with TRPV4 in lumbosacral DRG**. Confocal images of cryosections from *Htr3a*-EGFP transgenic animals stained with anti-TRPV4 are shown. All DRG presented are from lumbosacral axial levels. **(A–A”)** Sagittal section of 14 dpc fetal DRG. **(B–B”)** Sagittal section of 18 dpc fetal DRG. **(C–C”)** Coronal section of P2 DRG. **(D–D”)** Cryosection of P14 male DRG. **(E–E”)** Cryosection of P28 male DRG. All zoom insets are 400X.

Despite the known importance of TRPV4 in visceral nociception and adult bladder function (Cenac et al., 2008; Christianson et al., 2009; Everaerts et al., 2010; Franken et al., 2014; Yoshiyama et al., 2015), developmental expression patterns of this nociceptive marker in DRG have not previously been examined. To determine co-localization patterns of *Htr3a*-EGFP and TRPV4, we stained transgenic tissues for TRPV4. While we did observe positive staining in other known TRPV4+ tissues (kidney, bone, brain; data not shown), we did not detect TRPV4 in fetal or neonatal lumbosacral DRG (Figures 5A–C”). TRPV4 expression was detected in a subset of P14 and P28 DRG neurons; however, we did not observe overlap of TRPV4 immuno-staining with *Htr3a*-EGFP transgene expression at any of the stages examined (Figures 5D–E”).

### *HTR3a*-EGFP is expressed in a subset of myelinated sensory neurons

**Figure 6:**
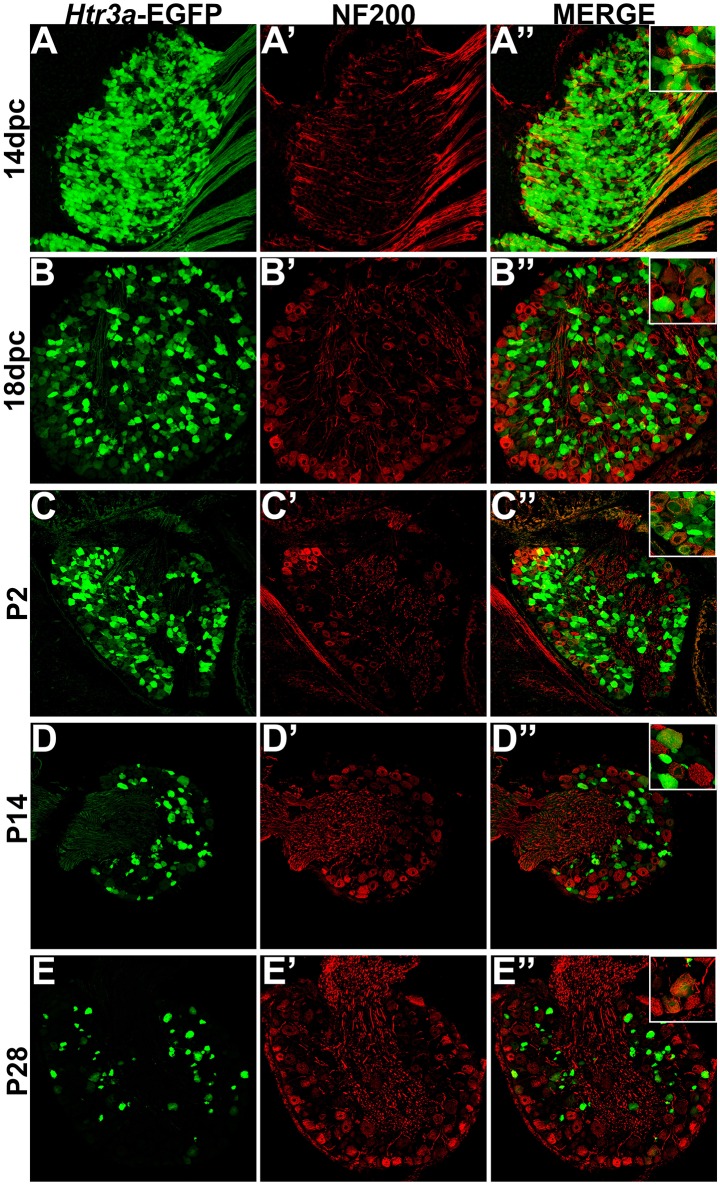
**A subset of *Htr3a*-EGFP neurons express Neurofilament 200**. Confocal images of cryosections from *Htr3a*-EGFP transgenic animals stained with anti-NF200 are shown. All DRG presented are from lumbosacral axial levels. **(A–A”)** Sagittal section of 14 dpc fetal DRG. **(B–B”)** Sagittal section of 18 dpc fetal DRG. **(C–C”)** Coronal section of P2 DRG. **(D–D”)** Cryosection of P14 male DRG. **(E–E”)** Cryosection of P28 male DRG. All zoom insets are 400X.

Following our characterization of *Htr3a*-EGFP in nociceptive C-fiber neurons, we next wanted to define patterns of overlap with mechanosensitive Aδ nociceptors. To this end we conducted immunohistochemical staining for Neurofilament 200 (NF200), a known marker of myelinated Aδ sensory neurons (Lawson et al., 1993). While expression of *Htr3a*-EGFP was prominent in the majority of cells in 14 dpc DRGs, NF200 was dim in a subset of cells and rarely overlapped with *Htr3a*-EGFP (Figures 6A–A”). By 18 dpc, however, many neurons expressed NF200 and some of these NF200+ cells also co-expressed *Htr3a*-EGFP (Figures 6B–B”). At P2, widespread co-expression of *Htr3a*-EGFP and NF200 occurred and was maintained at P14 and P28 (Figures 6C–E”).

### *Htr3a*-EGFP is expressed in the majority of bladder-innervating afferent neurons

**Figure 7:**
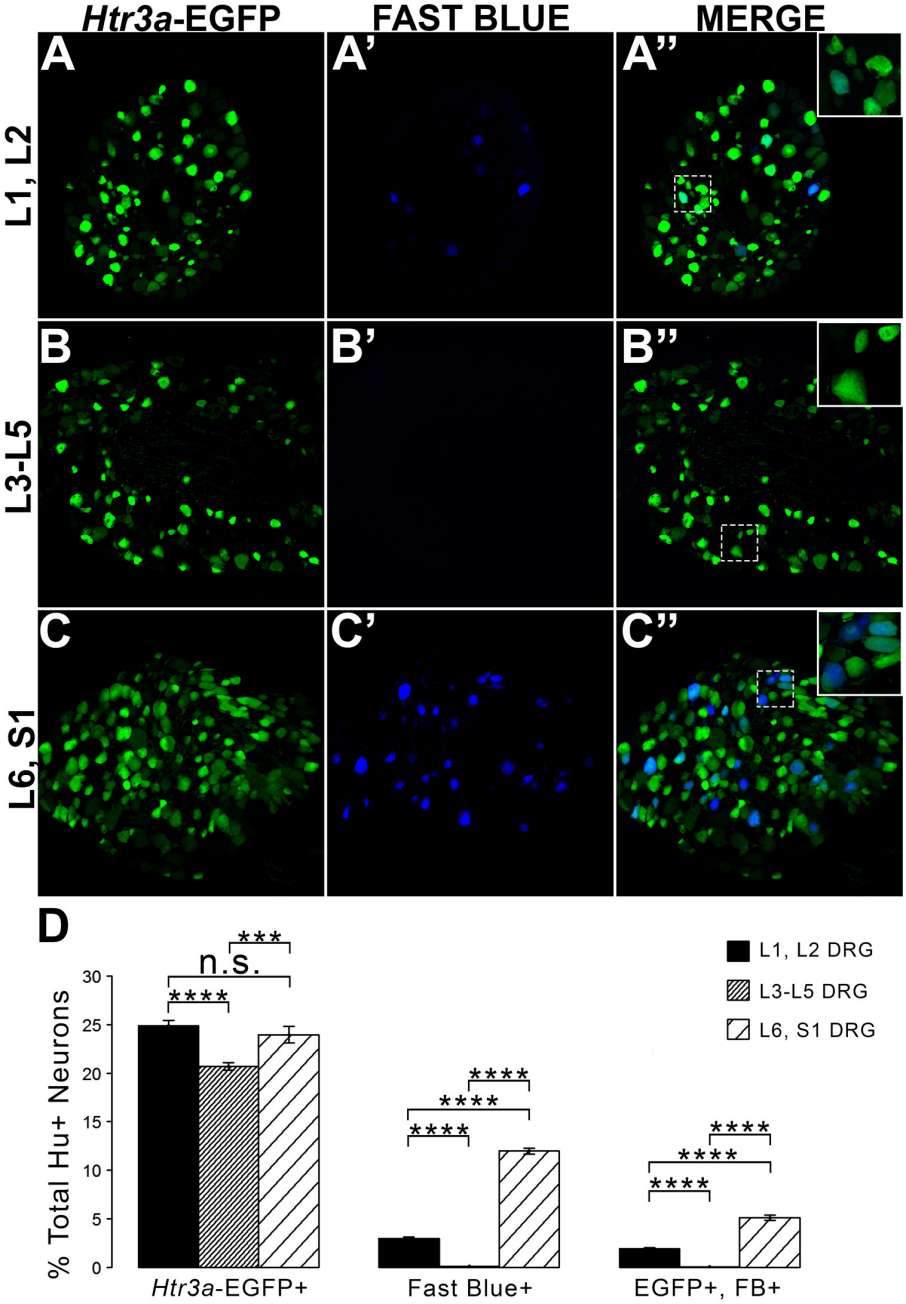
**L1, L2 and L6, S1 axial levels harbor bladder-projecting neurons**. Images of lumbosacral DRG harvested from *Htr3a*-EGFP transgenic male mice after retrograde tracing of bladder-projecting neurons are shown. **(A–A”)** L1, L2 axial level DRG sections. **(B–B”)** L3-L5 axial level DRG sections. **(C–C”)** L6, S1 axial level dorsal root ganglia sections. **(D)** Average percentages of Hu+ DRG neurons expressing the *Htr3a*-EGFP transgene, labeling with Fast Blue, and co-labeling with *Htr3a*-EGFP and Fast Blue at axial level groups L1, L2; L3-L5; and L6, S1. Error bars are standard error of the mean, *n* = 4 animals, 3–5 sections from 3 DRG quantified from each animal. ^***^p < 0.005, ^****^p < 0.001. Differences that are not significant (n.s.) are also indicated.

Given the known significance of 5-HT3A signaling in visceral nociception and neural control of bladder function (Zeitz et al., 2002; Bhattacharya et al., 2004; Faerber et al., 2007; Hall et al., 2015), we next sought to determine the proportion of 5-HT3A+ DRG neurons that contribute to bladder sensory innervation in adult mice. To do this, we conducted retrograde tracing of bladder innervation in adult male transgenic mice by injecting Fast Blue (FB) dye into the detrusor and quantified proportions of *Htr3a*-EGFP+ neurons whose soma were labeled with FB (Table 3 and Figure 7D). Among all three of the axial level groups, we noted significant differences in proportions of *Htr3a*-EGFP+ and FB+ neurons (*p* = 1.5564e-6 and *p* = 8.8e-16, respectively). At lumbar axial levels (L1, L2), nearly 25% of all Hu+ neurons express *Htr3a*-EGFP (Figures 7A–A”). Sacral axial level DRG neurons (L6, S1) are known to supply the majority of bladder sensory innervation, and we observed a similar proportion of *Htr3a*-EGFP expression in this population (23.9% of total L6, S1 Hu+ neurons expressed *Htr3a*-EGFP; Figures 7C–C”). Significantly fewer numbers of neurons within L3-L5 axial level DRGs, which do not contribute to bladder sensory innervation, showed *Htr3a*-EGFP+ expression (20.7% of Hu+ neurons were *Htr3a*-EGFP+, *p* = 7.168e-9 compared to L1, L2 and *p* = 0.002092 compared to L6, S1; Figures 7B–B”). When we quantified total numbers of neurons labeled by Fast Blue (FB+) retrograde tracing, we observed that nearly 3% of L1, L2 total neurons (Hu+) innervate the detrusor. In contrast, nearly 12% of L6, S1 neurons, a four-fold increase relative to lumbar levels, were labeled by Fast Blue in our experiments (*p* = 8.8e-16). We only very rarely observed any FB+ neurons in L3-L5 DRGs (0.067% of all Hu+ L3-L5 neurons; 26 out of 57,073 cells). The proportions of retrograde traced neurons in each of these axial levels are consistent with prior percentages reported for adult mice by other research groups (Keast and De Groat, 1992; Brumovsky et al., 2012).

**Table 3:** **Proportions of Total Neurons (Hu+) Expressing *Htr3a*-EGFP and Projecting to the Bladder (Fast Blue+)**.

	**L1, L2**	**L3-L5**	**L6, S1**
*Htr3a*-EGFP+/Hu+	24.89 ± 0.56%	20.68 ± 0.38%	23.97 ± 0.85%
Fast Blue+/Hu+	2.97 ± 0.14%	0.063 ± 0.025%	11.99 ± 0.30%
EGFP+, FB+/Hu+	1.92 ± 0.10%	0.027 ± 0.0099%	5.10 ± 0.28%
Total Hu+ Neurons			
Counted	47,208	57,073	40,415

**Figure 8:**
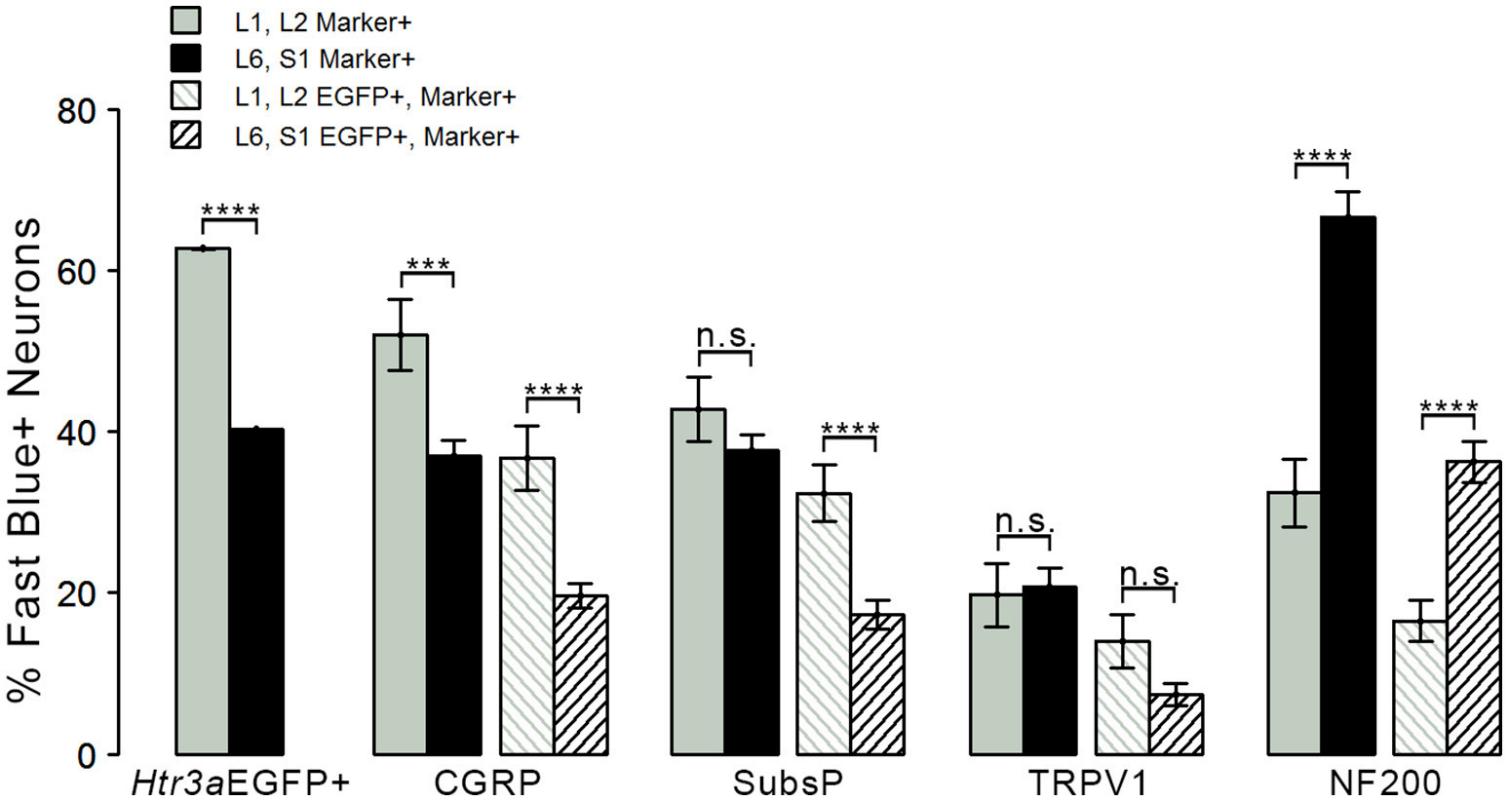
**Distinct expression patterns of 5-HT3A receptor in subclasses of bladder-projecting afferents**. Average percentages of bladder-projecting (Fast Blue+) neurons expressing the *Htr3a*-EGFP transgene and markers of sensory neuron subtypes in L1, L2 and L6, S1 DRG. Gray coloring represents L1, L2 proportions and black coloring represents L6, S1 proportions. *Htr3a*-EGFP average proportions in L1, L2 and L6, S1 DRG are the leftmost solid bars. Markers of sensory neuron subtypes CGRP, Substance P, TRPV1, NF200 (solid bars) and co-expression of *Htr3a*-EGFP and subtype markers (striped bars) are grouped by the marker used. Error bars are standard error of the mean, *n* = 4 animals, 3–5 sections from 3 DRG quantified from each animal. ^***^p < 0.005, ^****^p < 0.001. Differences that are not significant (n.s.) are also indicated.

Given that we had observed co-localization of *Htr3a* expression with markers of multiple types of nociceptive sensory neurons during development of lumbosacral DRG, we next sought to determine whether these subclasses of bladder-innervating neurons in adult mice maintain expression of the 5-HT3A receptor. To do this, we stained DRG sections from adult *Htr3a*-EGFP mice that had been processed for Fast Blue retrograde labeling of bladder projections for CGRP, Substance P, TRPV1, and NF200 (Figure 8 and Table 4). Of all bladder-innervating neurons at the L1, L2 axial levels (that is, all Fast Blue+ neurons), the majority of them express *Htr3a*-EGFP (62.8%). In more caudal DRG (L6, S1), where the majority of bladder sensory innervation originates, 40.3% of Fast Blue+ DRG neurons express *Htr3a*-EGFP. The difference in proportions of *Htr3a*-EGFP+, FB+ neurons in the lumbar and sacral axial levels was statistically significant (*p* = 4.349e-15). CGRP staining was observed in approximately half of all FB+ L1, L2 neurons (52.0%), while 36.9% of FB+ L6, S1 neurons were CGRP+ (*p* = 0.00292). In L1, L2 DRGs, 36.7% of FB+ neurons co-expressed *Htr3a*-EGFP and CGRP, but this proportion was reduced by half in L6, S1 neurons (19.6%) (*p* = 0.000162). When we stained for Substance P, we found similar expression levels in FB+ neurons in L1, L2 and L6, S1 DRG (42.8 vs. 37.7%, *p* = 0.251). However, we did observe a significant difference in proportions of FB+ neurons co-expressing *Htr3a*-EGFP and Substance P (32.4% of L1, L2 and 17.3% of L6, S1 FB+ neurons, *p* = 0.000310). Unlike the markers for peptidergic neurons, TRPV1 staining was nearly equivalent in FB+ neurons for both axial level groups; 19.7% of L1, L2 neurons were FB+ and 20.7% of L6, S1 neurons were labeled by Fast Blue (*p* = 0.8332). Despite the fact that proportions of TRPV1+ neurons were comparable in both lumbar and sacral bladder innervating DRG, there was a 2-fold difference in the numbers of *Htr3a*-EGFP+, TRPV1+ neurons in these locations. While 13.9% of TRPV1+ L1, L2 neurons exhibited co-expression of *Htr3a*-EGFP, only 7.4% of neurons were *Htr3a*-EGFP+, TRPV1+ among L6, S1 neurons. However, this difference did not reach statistical significance (*p* = 0.0741). Proportions of FB+ neurons that stained for NF200 were strikingly different from the other markers we examined. We found that 32.4% of FB+ L1, L2 neurons are NF200+, but this population was doubled in L6, S1 axial levels (66.6%) (*p* = 1.94e-8). NF200 and *Htr3a*-EGFP co-localize in 16.5% of FB+ L1, L2 neurons and in 36.3% of FB+ L6, S1 neurons (*p* = 7.544e-7). From these experiments we conclude that there are significant differences in the proportions of sensory neuron types that express the 5-HT3A receptor among the DRG groups that contribute to bladder innervation.

**Table 4:** **Proportions of Bladder-Projecting (Fast Blue+) Neuronal Subtypes in Lumbar (L1, L2) and Sacral (L6, S1) Axial Levels**.

	**Average Proportion in L1, L2**	**Marker+ out of Total FB+ Neurons Counted**	**Average Proportion in L6, S1**	**Marker + out of Total FB+ Neurons Counted**
*Htr3a*-EGFP+/FB+	62.75 ± 0.02%	938 out of 1431	40.29 ± 0.015%	1967 out of 4691
CGRP+/FB+	52.03 ± 4.46%	284 out of 463	36.96 ± 1.95%	494 out of 1340
EGFP+, CGRP+/FB+	36.73 ± 3.97%	198 out of 463	19.62 ± 1.51%	267 out of 1340
Subs P+/FB+	42.82 ± 3.96%	166 out of 387	37.66 ± 2.05%	464 out of 1240
EGFP+, Subs P+/FB+	32.34 ± 3.56%	124 out of 387	17.29 ± 1.75%	236 out of 1240
TRPV1+/FB+	19.74 ± 3.90%	56 out of 333	20.71 ± 2.45%	215 out of 1046
EGFP+, TRPV1+/FB+	13.99 ± 3.34%	40 out of 333	7.38 ± 1.42%	90 out of 1046
NF200+/FB+	32.42 ± 4.23%	90 out of 248	66.62 ± 3.19%	682 out of 1065
EGFP+, NF200+/FB+	16.49 ± 2.55%	51 out of 248	36.32 ± 2.54%	367 out of 1065

*Proportions of each marker or combination of markers were calculated for each DRG section and were then averaged. Averages are listed as percentages of total Fast Blue+ neurons plus or minus the standard error of the mean. Total numbers of neurons counted for each marker or combination of markers, out of all Fast Blue+ neurons counted, for all sections, is listed for L1, L2 and L6, S1 axial levels*.

**Figure 9:**
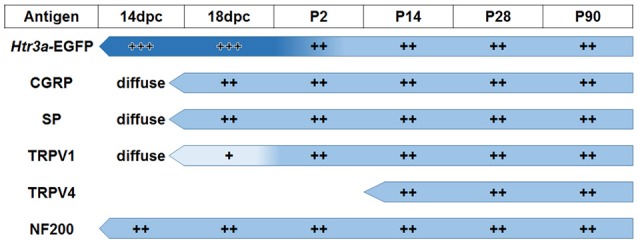
**Summary of developmental expression patterns in lumbosacral *Htr3a*-EGFP+ dorsal root ganglia neurons**. At 14 and 18 dpc, *Htr3a*-EGFP fluorescence is uniformly intense in the majority of lumbosacral DRG neurons. Fluorescence intensity diversifies at postnatal day 2, with some cells still strongly expressing the transgene while others are moderate or dim. This pattern is maintained through postnatal development and adulthood. Neuropeptides CGRP and Substance P are both extremely weak and diffuse at 14 dpc but their expression level increases by 18 dpc. The expression patterns observed for these markers at 18 dpc is maintained through adulthood. TRPV1 expression is negligible at fetal stages but escalates after birth at P2 and maintains expression levels. TRPV4 is not detected until P2 but is maintained from that time point to adulthood. NF200, unlike the other markers, is expressed strongly in the majority of neurons at 14 dpc, and its expression levels are stable through all time points examined.

The emerging expression patterns and intensity of expression for each of the markers we examined relative to *Htr3a* are graphically summarized in Figure 9.

## Discussion

Serotonin (5-hydroxytryptamine, 5-HT) is a known mediator of nociception, but contradictory effects are observed due to the wide variety of serotonin receptor subtypes expressed by nociceptors and their target tissues. One such receptor, 5-HT3A (encoded by *Htr3a* in mice), is a ligand-gated ion channel with previously established roles in potentiating pain (Zeitz et al., 2002; Hall et al., 2015). Despite its known expression in fetal neural crest derivatives (Tecott et al., 1993, 1995), comprehensive temporal profiling of 5-HT3A in developing sensory neuron populations has not been pursued. In this study we used the *Htr3a*-EGFP transgenic reporter line as a surrogate in combination with immunohistochemical labeling to identify neuron populations expressing 5-HT3A relative to a variety of nociceptive markers in normal fetal and postnatal DRG development.

We first noted expression of the *Htr3a*-EGFP transgene at 10 dpc in the neural tube and cranial ganglia, followed by up-regulation in lumbosacral DRG by 12 dpc. The early and sustained expression in sensory lineages suggests a functional role for 5-HT3A in differentiation of sensory neurons; however, the specific effects of this receptor in sensory neurogenesis and maturation remains to be determined.

Given that 5-HT3A is known to be involved in nociceptive processing (Zeitz et al., 2002), especially in visceral pain and inflammation (Randich et al., 2008; Kato, 2013), we sought to define the complement of nociceptive neuron subtypes that express the 5-HT3A receptor in development. Neuropeptides CGRP and Substance P are known effectors of inflammation and nociceptive processing (Laird et al., 2000; Saban et al., 2000; Kiss et al., 2001; Lagerström et al., 2011; Russell et al., 2014), but surprisingly their temporospatial expression patterns in development have not previously been reported. We found at 14 dpc both of these neuropeptides are detectable at low levels by immunohistochemistry in lumbosacral DRGs, while *Htr3a*-EGFP expression is much more widespread among neurons and is robustly transcribed. By 18 dpc, CGRP and Substance P expression is stronger and largely co-localizes with *Htr3a*-EGFP. In postnatal stages we noted the acquisition of a heterogeneous pattern of *Htr3a*-EGFP transgene expression, in which some neurons very strongly express *Htr3a*-EGFP while others show moderate or dim levels. Despite these changes in transgene expression, CGRP and Substance P expression patterns remained consistent from 18 dpc through P2, P14, and P28. Previous studies reported 5-HT3A receptor expression in “nonpeptidergic” sensory neurons based on relatively infrequent (4%) co-localization of 5-HT3A+ neurons identified by in situ hybridization with SP+ neurons that were detected by immunohistochemistry (Zeitz et al., 2002). In contrast, we found that the majority of SP+ neurons in developing and adult DRG expressed the *Htr3a*-EGFP transgene, indicating that at least some 5-HT3A+ populations are in fact peptidergic. The difference between our findings and prior reports maybe due to stages examined, DRG axial levels evaluated, greater ease of detecting EGFP fluorescence transgene vs. *in situ* hybridization signal, or differences between the mouse strains assayed.

TRP channels, namely TRPV1 and TRPV4, are key mediators of multimodal nociceptive processing in the lower urinary tract (Birder et al., 2002; Arms and Vizzard, 2011; Yoshiyama et al., 2015). Prior developmental studies of TRPV1 expression utilizing RT-PCR identified dynamic expression levels of this receptor over time, with low levels at 14 dpc (Hjerling-Leffler et al., 2007). While this finding could have been attributable to high expression in a few neurons with increasing numbers of neurons expressing the receptor over time, our immunohistochemical data shows that, in fact, TRPV1 is widely expressed at low levels through the DRG at 14 dpc. As lumbosacral DRG develop and mature, TRPV1 is expressed at higher levels in a relatively small proportion of total DRG neurons compared to *Htr3a*. The intensity of TRPV1 protein staining continues to increase and stabilizes by P2, with partial co-localization with *Htr3a*-EGFP in some DRG neurons. In marked contrast, TRPV4 protein is not detected until P14 and very rarely overlaps with *Htr3a*-EGFP transgene expression. This finding is especially interesting given that TRPV1 and TRPV4 have been implicated together in bladder afferent signaling (Janssen et al., 2016). The significant proportion of neurons co-expressing *Htr3a*-EGFP and NF200 indicates that 5-HT3A is not unique to a single nociceptive population, but is present in neurons of diverse sensory modalities and functions.

The second aim of our study was to determine the neurochemical diversity of 5-HT3A+ neurons that contribute to bladder sensory innervation. To this end we conducted retrograde tracing with Fast Blue dye in adult *Htr3a*-EGFP male mice and quantified proportions of 5-HT3A+ sensory neuronal subtypes projecting to the bladder. Co-staining DRG sections with nociceptive markers CGRP, SP, TRPV1, and NF200 revealed differences in proportions of *Htr3a*+ subtypes in lumbar (L1, L2) and sacral (L6, S1) groups of bladder-innervating DRG. The majority of Fast Blue+ lumbar neurons expressed *Htr3a*-EGFP, while less than half of the FB+ sacral neurons were EGFP+. This difference in expression may have a functional consequence; the majority of bladder muscle innervation is derived from the sacral DRG group (L6, S1) and these afferents are primarily stretch-sensitive mechanoreceptors expressing NF200 (Xu and Gebhart, 2008). This finding is especially interesting in the context of research conducted by other groups. In guinea pig *ex vivo* bladder preparations, direct application of serotonin resulted in significant excitation of stretch-sensitive bladder muscle afferents (Zagorodnyuk et al., 2009). Given the high proportion of sacral bladder afferents co-expressing *Htr3a* and NF200, these excitatory effects may be due to activation of this serotonin receptor. Future studies could determine if L6, S1 bladder afferents co-expressing *Htr3a* and NF200 also function to detect stretch of the bladder detrusor.

Given the large proportion of bladder-innervating neurons expressing *Htr3a* in male mice, 5-HT3A likely plays an important role in bladder afferent neural activity. In our retrograde tracing studies we focused only on male mice to avoid estrous cycles as a confounding factor in gene expression (Mónica Brauer and Smith, 2015). Since neurogenic bladder and bladder pain syndromes occur more frequently and with greater severity in women than men, sex-specific variation in gene expression may be a contributing factor to these differences (Irwin et al., 2006; Chrysanthopoulou and Doumouchtsis, 2014; Mónica Brauer and Smith, 2015). It will be of interest in subsequent studies to investigate sex differences in *Htr3a* gene expression patterns throughout development or into adulthood.

The substantial proportions of bladder-innervating afferents expressing 5-HT3A we identified were not unexpected, given prior reports examining the role of this receptor in micturition. Pharmacological activation of 5-HT3A at spinal and supraspinal terminals resulted in inhibited sensory processing and a decreased micturition threshold volume (Espey et al., 1998). Complementary experiments showed that intrathecal administration of a 5-HT3A antagonist led to bladder nociceptive hypersensitivity (Hall et al., 2015), further supporting a critical role for 5-HT3A in sensory mediation of bladder filling and voiding. Future studies will need to focus on elucidating the specific functional impact of this receptor on bladder contractility and the consequences of its absence during nervous system development.

Overall our work is the first to delineate the expression patterns of a well-studied serotonin receptor in the understudied system of bladder innervation in the context of sensory neuron development. The work presented here serves as a foundation for future studies focusing on the contribution of the 5-HT3A receptor to common disorders such as chronic pelvic pain and urinary incontinence.

## Author contributions

KER performed acquisition of data; analysis and interpretation of data; statistical analysis, and drafting of the manuscript. EMS^2^ designed and supervised the study, participated in interpretation of data, critically revised the manuscript for important intellectual content, and obtained funding.

### Conflict of interest statement

The authors declare that the research was conducted in the absence of any commercial or financial relationships that could be construed as a potential conflict of interest.
